# Genome-Wide Expression Analysis in Down Syndrome: Insight into Immunodeficiency

**DOI:** 10.1371/journal.pone.0049130

**Published:** 2012-11-14

**Authors:** Chong Li, Lei Jin, Yun Bai, Qimin Chen, Lijun Fu, Minjun Yang, Huasheng Xiao, Guoping Zhao, Shengyue Wang

**Affiliations:** 1 School of Life Science, Fudan University, Shanghai, China; 2 Shanghai-MOST Key Laboratory of Health and Disease Genomics, Chinese National Human Genome Center at Shanghai, Shanghai, China; 3 Shanghai Children's Medical Center, Shanghai Jiao Tong University School of Medicine, Shanghai, China; 4 National Engineering Center for Biochip at Shanghai, Shanghai, China; The Ohio State Unversity, United States of America

## Abstract

Down syndrome (DS) is caused by triplication of Human chromosome 21 (Hsa21) and associated with an array of deleterious phenotypes, including mental retardation, heart defects and immunodeficiency. Genome-wide expression patterns of uncultured peripheral blood cells are useful to understanding of DS-associated immune dysfunction. We used a Human Exon microarray to characterize gene expression in uncultured peripheral blood cells derived from DS individuals and age-matched controls from two age groups: neonate (N) and child (C). A total of 174 transcript clusters (gene-level) with eight located on Hsa21 in N group and 383 transcript clusters including 56 on Hsa21 in C group were significantly dysregulated in DS individuals. Microarray data were validated by quantitative polymerase chain reaction. Functional analysis revealed that the dysregulated genes in DS were significantly enriched in two and six KEGG pathways in N and C group, respectively. These pathways included leukocyte trans-endothelial migration, B cell receptor signaling pathway and primary immunodeficiency, etc., which causally implicated dysfunctional immunity in DS. Our results provided a comprehensive picture of gene expression patterns in DS at the two developmental stages and pointed towards candidate genes and molecular pathways potentially associated with the immune dysfunction in DS.

## Introduction

Down syndrome (DS; trisomy 21) is characterized by a complete, or occasionally partial, triplication of Hsa21. With an incidence of about one in 750 births [1], DS is the most common autosomal abnormality affecting live-born infants. More than 80 clinical features with variation in number and in severity are reported in DS [2].

DS patients are clinically associated with multiple blood cell-related phenotypes, including increased risk to develop leukemia, decreased lymphocyte counts, markedly enhanced incidence of autoimmune disorders as well as vulnerability to recurrent bacterial and viral infections [3], [4], [5], [6]. Moreover, pneumonia and other types of respiratory infections are the most common causes of death in DS children and early adults [7]. The abnormalities highlight that DS individuals are very likely associated with intrinsic defects of the immune system [8]. However, the molecular mechanisms by which trisomy 21 leads to the immune system disorders in DS remain poorly investigated. Transcriptome of peripheral blood cells from DS would provide a unique molecular window into immunodeficiency relevant to DS. Several gene-expression studies in T lymphocytes [9] and blood cells [10] from DS patients reported dysregulated expression of some immune-associated genes, yet very small sample size or age-unmatched controls restricted statistical analysis.

Here, we characterized gene-expression in uncultured blood cells from DS and age-matched control samples in neonate and child group. The dysregulated genes in DS at the two developmental stages were identified and showed a comprehensive picture of gene expression patterns. Furthermore, classification of dysregulated genes based on their known functions provided insight into immunodeficiency in DS patients.

## Results

### Identification of differentially expressed genes between DS and control samples

Using the Affymetrix GeneChip Human Exon 1.0 ST Array containing ∼20,000 known human genes, we performed gene expression analysis in DS peripheral blood cells and age-matched controls from N and C group. Of 17,626 core transcript clusters with RefSeq-supported annotation, 13,027 in C group and 13,168 in N group (corresponding to 12,876 and 13,017 well-characterized genes, respectively) were reliably expressed. In RNA-Seq analysis of endothelial progenitor cells from DS and control, 13,144 active RefSeq genes were shared by both the cells [11]. This suggests that the number of the expressed genes in DS and control cells was comparable in the two studies.

**Figure 1:**
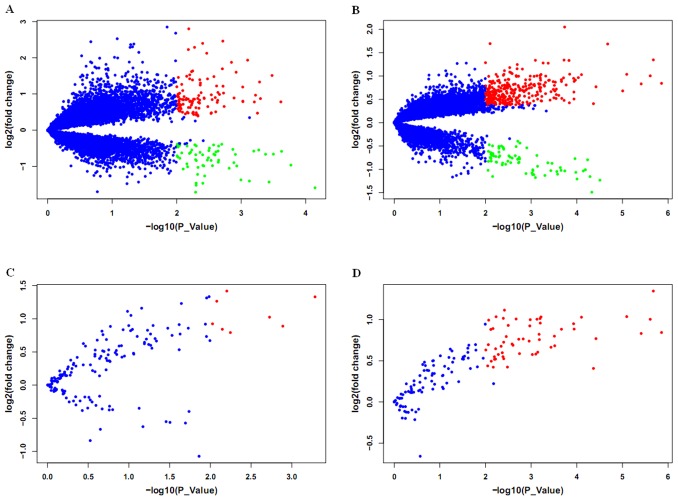
A global and local view of expression patterns in DS in the two age groups. Each dot represents a gene. Red and green dots indicate genes significantly up- and down-regulated in the DS samples, respectively. Blue dots indicate genes without change. (A) and (B) represent the expressed transcript clusters on whole genome in N and C group, respectively. (C) and (D) represent the expressed transcript clusters located on Hsa21 in N and C group, respectively.

To identify differentially expressed genes between DS and controls in each group, we used ANOVA in combination with fold change in gene expression. Consistent with the observations of Prandini et al. [12], gender and DS×gender effects were not significant for expressed Hsa21 genes in DS individuals; however, expression of some non-Hsa21 genes were significantly affected by the two effects in each group. Therefore, the two effects were kept in our analysis. In N group, 174 (1.3%) of the 13,168 transcript clusters, containing 113 (64.9%) up-regulated genes and 61 (35.1%) down-regulated genes, were differentially expressed between DS and controls (Figure 1A, Table S1). In C group, 383 (2.9%) transcript clusters displayed differential expression between DS and controls. Pearson's correlation test showed no significant results between the child age and expression of the genes (smallest q value was 0.99). Among the 383 transcript clusters, 312 (81.5%) were up-regulated and 71 (18.5%) were down-regulated in DS children (Figure 1B, Table S2). The higher percentage of up-regulated genes in N and C group was similar to prior report [13]. In the two groups, only 22 dysregulated genes, including six transcript clusters on Hsa21, were shared (Table 1). Elevated expression of the six transcript clusters was also implicated in other DS cells or tissue (Table 1). For the 22 transcript clusters, the extent of fold change between the two groups was significantly correlated (Pearson's cor = 0.92, P = 1.28×10^−9^), suggesting their importance in DS phenotypes at the two developmental stages.

**Table 1:** The common dysregulated genes in the two age groups.

Gene symbol	Gene description	N group		C group		Chr.	Reference^b^
		Ratio	P	Ratio	P		
*NAV1*	neuron navigator 1	1.45	9.1E-03	1.35	4.7E-03	chr1	no
*RALGPS2*	Ral GEF with PH domain and SH3 binding motif 2	0.42	3.7E-03	0.43	9.9E-05	chr1	no
*AFF3*	AF4/FMR2 family, member 3	0.39	1.0E-03	0.61	6.6E-03	chr2	no
*OSBPL10*	oxysterol binding protein-like 10	0.33	1.0E-04	0.43	3.1E-05	chr3	no
*TLR10*	toll-like receptor 10	0.36	5.0E-03	0.46	9.0E-04	chr4	no
*HLA-DOA*	major histocompatibility complex, class II, DO alpha	0.50	1.2E-03	0.61	7.0E-04	chr6	no
*HLA-DOB*	major histocompatibility complex, class II, DO beta	0.43	4.7E-03	0.49	1.0E-04	chr6	no
*PLXNA4*	plexin A4	1.77	9.3E-03	1.60	6.0E-04	chr7	no
*NPDC1*	neural proliferation, differentiation and control, 1	1.50	9.6E-03	1.60	3.0E-04	chr9	no
*PAX5*	paired box 5	0.36	3.9E-03	0.55	1.9E-03	chr9	no
*C13orf18*	chromosome 13 open reading frame 18	0.49	4.7E-03	0.47	1.3E-03	chr13	no
*P2RX5*	purinergic receptor P2X, ligand-gated ion channel, 5	0.50	3.8E-03	0.63	6.9E-03	chr17	no
*CD22*	CD22 molecule	0.30	5.1E-03	0.49	2.0E-04	chr19	no
*CLEC17A*	C-type lectin domain family 17, member A	0.38	7.0E-04	0.55	4.0E-04	chr19	no
*CST7*	cystatin F (leukocystatin)	2.74	9.4E-03	1.80	6.3E-03	chr20	no
*ATGAT3* a	1-acylglycerol-3-phosphate O-acyltransferase 3	1.73	5.7E-03	2.05	8.1E-06	chr21	[12]
*ATGAT3* a	1-acylglycerol-3-phosphate O-acyltransferase 3	1.90	9.4E-03	1.79	1.4E-06	chr21	[12]
*ITGB2*	integrin, beta 2	2.03	1.9E-03	1.73	3.5E-03	chr21	[12], [13],
*PDXK*	pyridoxal (pyridoxine, vitamin B6) kinase	2.51	5.0E-04	1.93	1.0E-04	chr21	[14], [16]
*PTTG1IP*	pituitary tumor-transforming 1 interacting protein	1.79	7.1E-03	1.46	6.4E-03	chr21	[13], [14], [16]
*TRPM2*	transient receptor potential cation channel, subfamily M, member 2	2.67	6.3E-03	1.51	4.3E-03	chr21	[12]
*SLC9A7*	solute carrier family 9 (sodium/hydrogen exchanger), member 7	0.52	6.9E-03	0.69	5.1E-03	chrX	no

a Different transcript clusters of the same gene.

b Reference detecting dysregulation of the gene in DS studies.

**Figure 2:**
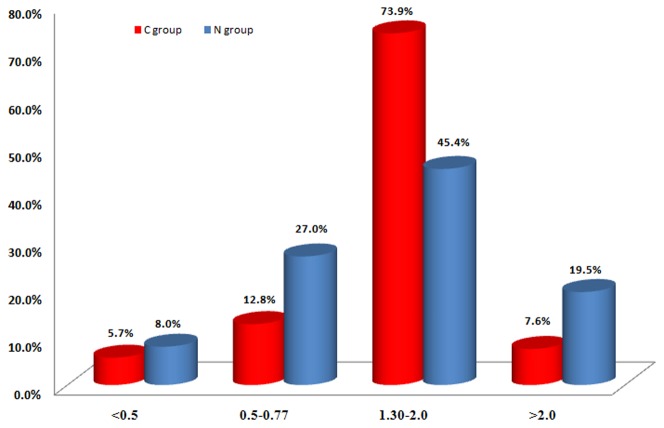
Fold change (DS/control) distribution of the dysregulated genes in DS. The x-axis indicates fold changes which are divided into four intervals and the y-axis represents percentage of the genes in one interval in all the dysregulated genes in one group.

Furthermore, the dysregulated genes in each group were divided into four intervals according to fold change (Figure 2). The genes that were mildly altered in expression, with fold change between 0.5∼0.77 or 1.3∼2.0, accounted for the majority of the dysregulated genes in each group. The genes whose expression was intensively varied (fold change <0.5 or >2) were in the minority.

### Expression variation of Hsa21 genes in DS

Given trisomy of Hsa21, we assessed expression variation of genes on this chromosome. There are 237 transcript clusters on the array, corresponding to 234 known genes on Hsa21. Of the transcript clusters, 157 (66%) and 146 (62%) were expressed in N and C group, respectively. There were 5.1% (8/157) in N group and 38.3% (56/146) in C group with significant expression difference between DS and age-matched controls (Figure 1C, D). Of the expressed Hsa21 genes in the two groups, no significantly down-regulated genes was detected in DS samples (Figure 1C and D), which is consistent with other reports [12], [14] and reflects the dosage effects of trisomy. The average expression ratios (DS/control) of expressed Hsa21 genes were 1.29±0.43 (mean ± sd) in N group and 1.40±0.35 in C group, revealing an overall up-regulation of Hsa21 genes which is also observed in previous data [12], [15], [16], [17].

### QPCR validation

**Figure 3:**
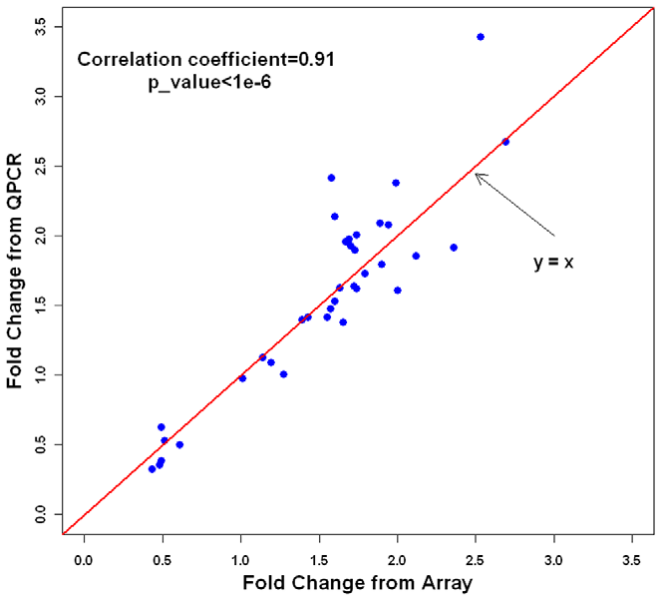
Comparison of fold change between array and QPCR. A blue dot represents a gene.

To confirm changes in gene-expression levels detected by the arrays and assess our statistical methods, we conducted approximately 2,500 QPCRs of all 37 cDNA samples from DS and control individuals. We first checked our definitions about a reliably expressed gene. To this end, several genes, whose normalized expression signals on the array in some individuals were close to the threshold according to our definition, were analyzed in the assay. These genes included *VAV2* in N group and *BTG3*, *PDGFD* and *PDGFRB* in C group. Their signals, albeit very weak relative to the endogenous genes, were detected by QPCR in all samples, thereby indicating the reasonable threshold defined by our criterions. We next selected a subset of the genes from each group and measured their expression levels. These genes included Hsa21 and non-Hsa21 genes that were statistically up-regulated, unaltered or down-regulated in analysis of the array. DS/control ratio for each gene was calculated and was very consistent with fold change obtained from the array (Table S3, Figure S1), with a Pearson correlation coefficient of 0.91 (P = 2.2×10^−14^) (Figure 3).

### Chromosomal distribution of the dysregulated genes

**Figure 4:**
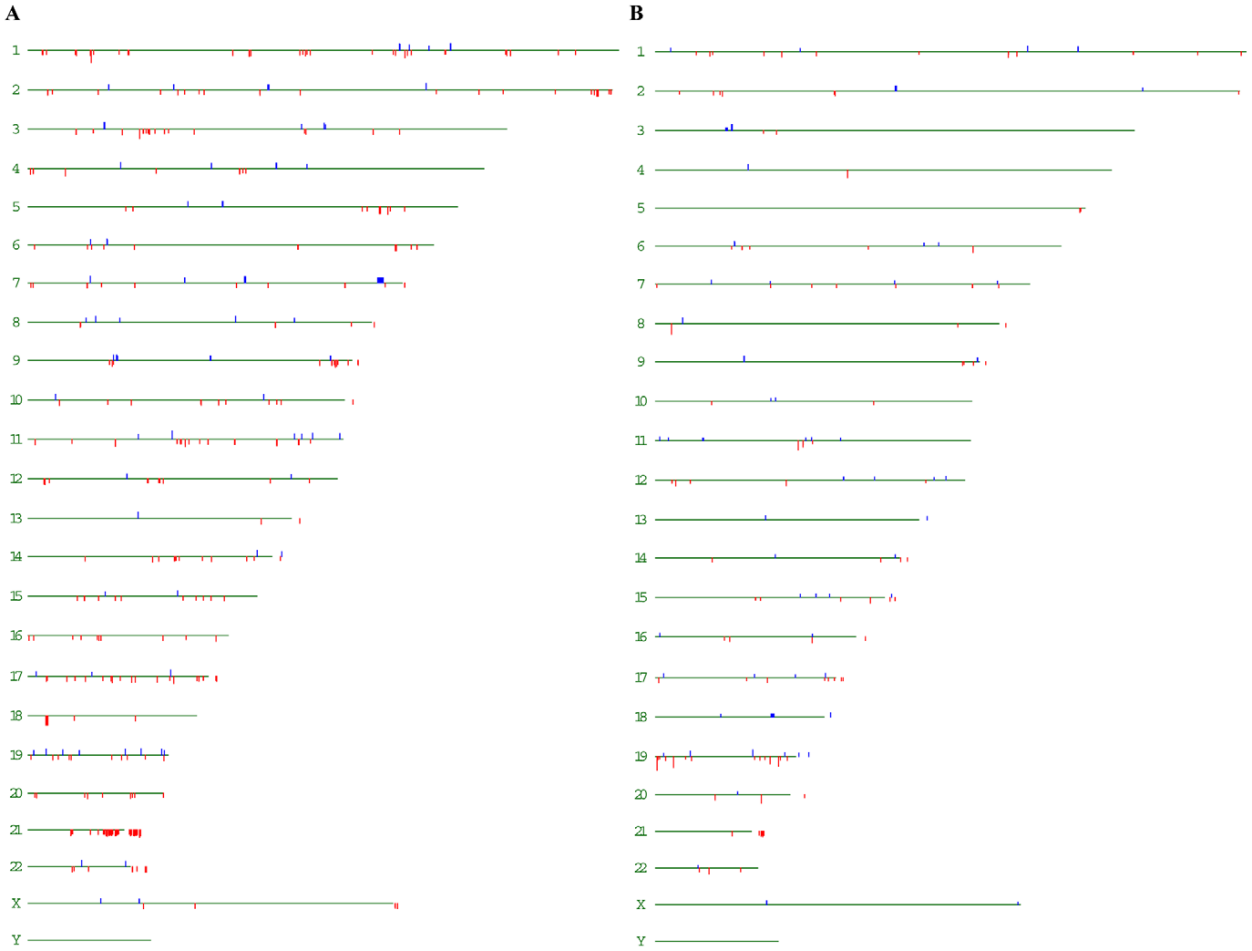
Chromosomal distribution of the differentially expressed genes. (A) and (B) indicate the chromosomal distribution of the 174 and 383 differentially expressed transcript clusters in N and C group between DS and age-matched controls, respectively. Blue bars represent lower expressed genes and red bars represent higher expressed genes in DS individuals.

**Figure 5:**
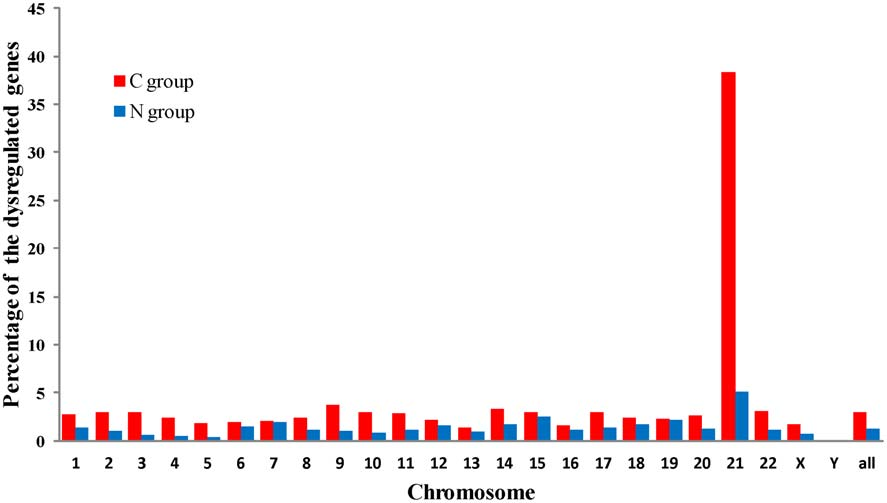
Percentage of dysregulated genes in expressed genes. Each bar indicates the percentage of the dysregulated genes in the expressed genes on each chromosome. “all” represents the percentage of total dysregulated genes in all expressed genes in each group.

We analyzed chromosomal distribution of the dysregulated genes in N and C group to evaluate effects of trisomy 21 on the whole genome. Of the dysregulated transcript clusters, 326 and 166 were mapped to non-Hsa21 in N and C group, respectively. These data are in agreement with the prior data [13], [15], [18], providing further evidence for the idea that both expression changes of Hsa21 and non-Hsa21 genes contribute to the etiology of DS. Chromosomal distribution of these dysregulated genes in N group and C group were showed in Figure 4. Figure 5 illustrated the percentage of the dysregulated genes in the expressed genes at individual chromosome and the whole genome level (corresponding to “all”). Through binomial tests, chromosome 21 in each group was significantly overrepresented with Pc = 0.027 in N group and Pc<10^−6^ in C group after Benjamini-Hochberg (BH) correction [19]. None of the remaining chromosomes was significantly overrepresented or underrepresented at Pc<0.05.

### Hierarchical cluster analysis of the dysregulated genes

**Figure 6:**
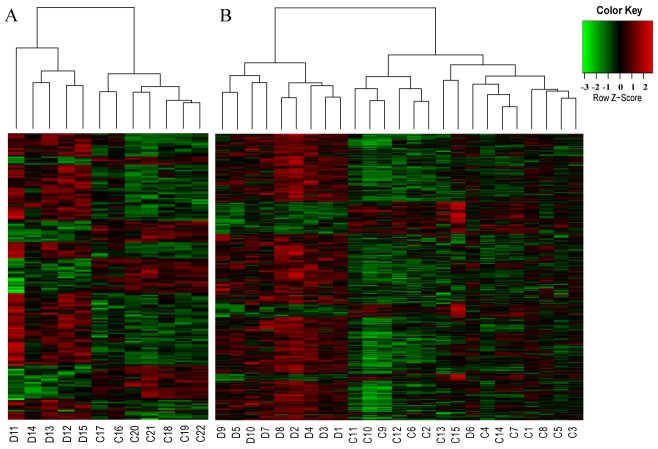
Hierarchical clustering of the differentially expressed genes. (A) hierarchical clustering of N group (DS: D11–D15 versus control: C16–C22) based on the 174 dysregulated transcript clusters (rows). (B) hierarchical clustering of C group (DS: D1–D10 versus control: C1–C15) based on the 383 differentially expressed transcript clusters. Red indicates higher expression and green indicates lower expression.

In Figure 6, we illustrated the results of hierarchical clustering on the dysregulated transcript clusters in DS. Cluster analysis clearly separated the DS neonates from controls (Figure 6A). The child samples were divided into two major distinguishable groups, leaving one DS exception (D6) grouped into the control group (Figure 6B). This exception is not surprising, because although some phenotypes frequently occur in DS patients, the degree to which individual is affected varies. It is likely that the misclassification of the DS child is a reflection of mild phenotypic abnormalities caused by a combination of environmental and genetic variation.

We further asked whether hierarchical clustering could distinguish DS from control individuals based on the dysregulated non-Hsa21 genes. DS neonates were also separated from the matched controls (Figure S2A), which is similar to the observation that hierarchical clustering distinguishes DS from control fetuses based on the non-Hsa21 altered genes in amniotic fluid cell-free mRNA [18]. Unlike N group, DS and control children were clearly divided into three groups (Figure S2B). The first group included 11 controls and sample D6 that showed different expression patterns from the other DS samples in Figure 6B. The second group included the remaining 9 DS samples. The third group, including control C1, 3, 5 and 8, seemed to display intermediate patterns between the other two groups. The four controls were also grouped together as shown in Figure 6B, showing less well-defined expression patterns in these samples.

### Effects of transcription factors on disruption of non-Hsa21 genes in DS

The dysregulated non-Hsa21 genes in each group demonstrated the pervasive effects of trisomy 21 on the whole genome. One hypothesis suggests that disruption of non-Hsa21 genes in DS is through modulation of transcription factors (TFs) [20]. Therefore, we searched the dysregulated genes in each group for TFs. Expectedly, ten TFs in C group and two in N group were deregulated (Table 2). We next asked whether targets of the TFs were accordingly altered in expression. We first checked *Pax5* which was down-regulated in both of the groups. Six genes, *CD19*, *CD79A*, *BLNK*, *EBF1*, *FCER2* and *SPIB*, are activated by *Pax5* [21], [22], [23]. Expression of these targets was down-regulated in DS children and neonates, however, only *CD79A*, *BLNK*, *FCER2* and *SPIB* were expressed with significant alteration in DS children (Table S6). We further examined expression pattern of the known targets of the other TFs in our array data. A total of 31 genes can be activated or repressed by the TFs (Table S6); nonetheless, expression of the targets was not significantly altered accordingly. The reason for this could be that dysregulation of target gene expression also depends on other deregulated TFs or coactivators. These data indicate that deregulation of these TFs plays limited roles in massive disruption of non-Hsa21 gene expression in DS.

**Table 2:** The dysregulated transcription factors in DS.

Accession number	Gene symbol	Gene description	Ratio (DS/control)	P_value (ANOVA)	Chromosome	Group
NM_005263	*GFT1*	growth factor independent 1 transcription repressor	1.82	2.9E-04	chr1	C
NM_001040667	*HSF4*	heat shock transcription factor 4	1.38	6.0E-04	chr16	C
NM_003121	*SPIB*	Spi-B transcription factor (Spi-1/PU.1 related)	0.49	9.1E-04	chr19	C
NM_001001890	*RUNX1*	runt-related transcription factor 1	1.50	1.8E-03	chr21	C
NM_002040	*GABPA*	GA binding protein transcription factor, alpha subunit 60 kDa	1.55	9.8E-03	chr21	C
NM_001951	*E2F5*	E2F transcription factor 5, p130-binding	0.49	8.7E-03	chr8	C
NM_016734	*PAX5*	paired box 5	0.55	1.9E-03	chr9	C
NM_022465	*IKZF4*	IKAROS family zinc finger 4 (Eos)	1.34	8.3E-03	chr12	C
NM_015995	*KLF13*	Kruppel-like factor 13	1.51	7.2E-03	chr15	C
NM_001031804	*MAF*	v-maf musculoaponeurotic fibrosarcoma oncogene homolog (avian)	1.99	8.6E-04	chr16	C
NM_198182	*GRHL1*	grainyhead-like 1 (Drosophila)	1.39	2.9E-03	chr2	N
NM_016734	*PAX5*	paired box 5	0.36	3.9E-03	chr9	N

### Functional analysis of the dysregulated genes

To explore biological functions of the dysregulated genes in each group, we analyzed the biological process and KEGG pathways through Onto-tools. The significantly enriched GO biological processes and KEGG pathways (Pc<0.05 after BH correction) were showed in Table 3. One GO biological process and two KEGG pathways were found to be enriched in N group. Likewise, one GO biological process and six KEGG pathways were enriched in C group.

**Table 3:** Enriched GO biological processes and KEGG pathways in the dysregulated genes in DS.

Age group	GO/KEGG ID	Biological Process/KEGG Pathway	Gene Symbol	P^a^	Pc^b^
Neonate group	GO: 45087	innate immune respones	*C1QC; FCGR1A; PGLYRP1; C1R; TLR10; CYBA; AKIRIN2*	<1.0E-04	9.3E-04
	KEGG: 05322	Systemic lupus erythematosus	*HLA-DOA; HLA-DOB; C1R; C1QC; FCGR1A; ELA2*	2.0E-04	9.0E-03
	KEGG: 04670	Leukocyte transendothelial migration	*CYBA; ITGB2; MAPK14; VAV2; MMP9*	1.1E-03	2.4E-02
Child group	GO: 7159	leukocyte adhesion	*CERCAM; ITGAL; ITGB2; ITGB1; EZR*	<1.0E-05	6.5E-03
	KEGG: 04510	Focal adhesion	*PDGFRB; MAP2K1; FYN; ITGA10; DIAPH1; COL6A2; PDGFD; CAPN2; SHC1; LAMB2; PPP1CA; ITGAV; ERBB2; ITGB1*	1.6E-06	6.9E-05
	KEGG: 04514	Cell adhesion molecules (CAMs)	*ITGB1; ITGB2; ITGAV; NCAM1; ITGAL; CD22; HLA-DOA; PTPRM; SPN; HLA-DOB; CNTAP2*	2.0E-06	6.9E-05
	KEGG: 04810	Regulation of actin cytoskeleton	*ITGB1; ITGB2; ITGAV; ITGAL; TIAM1; PPP1CA; PDGFRB; PDGFD; EZR; MAP2K1; DIAPH1; ITGA10*	6.6E-05	1.6E-03
	KEGG: 04662	B cell receptor signaling pathway	*CD79A; RASGRP3; BLNK; CD22B; CD78B; CD72*	4.0E-04	6.2E-03
	KEGG: 05340	Primary immunodeficiency	*BLNK; CD79A; TNFRSF13C; ADA*	1.6E-03	2.2E-02
	KEGG: 04650	Natural killer cell mediated cytotoxicity	*PRF1; MAP2K1; SHC1; CD244; FYN; ITGB2; ITGAL*	2.4E-03	2.8E-02

a Nominal p values.

b Adjusted p values after BH correction.

## Discussion

In present study, we analyzed gene expression difference between uncultured blood cells from DS and controls at the two age stages. A total of 383 and 174 dysregulated transcripts were identified in DS children and neonates, respectively and the array data were validated by approximately 2,500 QPCRs. Using functional profiling analysis, we identified significantly disrupted biological pathways which were relevant to immunodeficiency observed in DS.

The frequency (35.1%) of the down-regulated genes in DS neonates was higher than that (18.5%) in DS children. In amniotic fluid cell-free mRNA [18] and heart tissue [24] from DS fetuses, 46% and 59% of the dysregulated genes showed down-regulation, respectively. However, the reason for these observations is unclear. Given that TFs usually can activate or repress multiple targets, we postulated that the deregulated TFs exclusively in N or C group could contribute to the frequency difference observed in our study. *GRHL1* expression was up-regulated exclusively in DS neonates, however, its target, *P450scc* [25], showed normal expression. Similarly, the known targets of the altered TFs only in DS children were normally expressed (Table S6). The data point to limited contribution of TFs to the frequency difference between N and C group. Other mechanism responsible for the observations remains further postulated.

In DS neonates, only eight transcript clusters on Hsa21 were significantly up-regulated in expression. The finding is compatible with the previous data that a quite small number of Hsa21 genes are dysregulated in DS fetus samples [16], [18], [26]. We thus hypothesize that triplication of Hsa21 *per se* induces a modest dysregulation of Hsa21 genes during early developmental stages. The modest dysregulation might partially account for the phenotypes of DS individuals and mouse models at early stages. It has been reported that the basal forebrain cholinergic system is apparently normal in DS fetuses and infants, based on both neuronal numbers and choline acetyltransferase activity [27], [28]. In Ts65Dn mice, the cerebellum has normal size at birth as compared to wild type littermates [29].

We analyzed functions of the 22 common dysregulated genes in both age groups. Among the 22 genes, *ITGB2*, *HLA-DOA*, *HLA-DOB*, *Pax5* and *CD22* are more associated with the immune system based on published data. *ITGB2* encodes β chain of the integrin lymphocyte functional antigen-1 (*LFA-1*) and its overexpression increases aggregation of DS lymphoblastoid cell lines (LCLs) [30]. *HLA-DOA* and *HLA-DOB* form HLA-DO which functions as a modulator of Ag presentation [31]. Their down-regulation in DS might affect efficiency of Ag presentation. Both *Pax5* and *CD22* were down-regulated in DS children and neonates. *Pax5* plays an essential role in B-lineage commitment [32]. Strikingly, *Pax5*(−/−) cells show slower growth, decreased surface IgM expression, and total loss of B cell receptor signaling [33]. *CD22*-deficient mice exhibits a reduced number of mature B cells in circulation and a significant diminution of surface Ig levels in these B cell subpopulations [34], [35]. Deregulation of the five genes suggests their critical roles in immunodeficiency in DS patients.

We further explored biological functions of the dysregulated genes in the two age groups at a genome-wide level. In N group, innate immune response and two KEGG pathways, systemic lupus erythematosus (SLE) and leukocyte trans-endothelial migration, were enriched. The process or pathways are directly linked to the immune system, thereby supporting the hypothesis that the immune system in DS is intrinsically deficient from the very beginning [8]. Leukocyte trans-endothelial migration is vital for immune surveillance and inflammation [36]. In this pathway, *Vav2* expression in DS neonates was down-regulated (Table S3). The gene is an activator of *Cdc42*, *Rac1* and *RhoA* [37] which regulate actin dynamics and gene expression. Down-regulation of *Vav2* could disturb leukocyte migration, for knockdown of *Vav2* prevents *Rac* activation in some cells [38].

In C group, one biological process and six pathways were significantly deregulated in PBMCs of DS patients. In the process of leukocyte adhesion, overexpression of *ITGB2* and *ITGAL* whose products form integrin LAF-1 has been reported in LCLs from DS patients and increase adhesiveness of these cells [30]. It is worthy of note that product of *ITGB1* can form 11 heterodimeric integrins through pairing with alpha chain of integrins [39]. Up-regulated expression of the gene could have an impact on balance of the associated integrins. The first three pathways are focal adhesion, cell adhesion molecules (CAMs) and regulation of actin cytoskeleton which are implicated in cell growth, survival and mobility [40], [41],[42]. It has been shown that absolute total lymphocytes are significantly lower in DS children than controls [43], [44], [45]. Also, T lymphocyte maturation is impaired in DS individuals [46]. Our findings could be used to evaluate these observations associated with DS. The three remaining pathways are B cell receptor signaling pathway, primary immunodeficiency and natural killer cell mediated cytotoxicity. Individuals with DS display increased susceptibility to recurrent infections [4] and significantly reduced IgG2 levels [45]. Moreover, lower NK cytotoxic activity compared to controls has been observed in DS children and adults [47], [48]. Disturbance of the three pathways could contribute to immune dysfunction observed in DS.

There are several potential limitations in this work. One limitation is that total RNA was isolated from distinct cell types and cell composition between N and C group. Another is smaller sample size in N group which could reduce statistical power. For a more comprehensive analysis of gene expression in DS, one would need to consider cell type difference and sample size.

## Materials and Methods

### Ethics statement

The Ethical Committee of the Chinese National Human Genome Center at Shanghai approved this project for the involvement of human subjects (approval ID: 201201) and parents of all the individuals provided written informed consent.

### Samples and groups

All samples, including 15 DS patients and 22 controls, were collected at the Shanghai Children's Medical Centre from peripheral blood of the participants. All DS patients were confirmed by karyotyping. The samples were divided into two groups: N group (5 DS versus 7 control individuals, age: 3 days to 38 days) and C group (10 DS versus 15 control individuals, age: 1 year to 13 years). In each group, age and cell type were matched between DS and controls. Although the mean age of all the DS individuals was less than that of all control individuals, the difference was not statistically significant (p = 0.27 and p = 0.20 for C and N group, respectively, Student's t test) (Table S4).

### Total RNA isolation

From the 25 child samples (C group), peripheral blood mononuclear cells (PBMCs) were isolated from peripheral blood using Lympholyte ®-H (CEDARLANE), and total RNA was extracted using the mirVana™ miRNA Isolation Kit (Invitrogen) according to the manufacturer's protocol. The remaining 12 neonate samples (N group) had limited blood volume; therefore, total RNA was extracted from peripheral blood cells (PBCs). RNA concentration and purity was tested using a Nanodrop 2000c spectrophotometer (Thermo Scientific). RNA quality was further assessed with the Agilent 2100 Bioanalyzer using RNA 6000 Nanochips (Agilent Technologies). None of the 37 RNA samples had DNA contamination or RNA degradation. RNA samples were stored at −80°C for hybridization.

### Screening of *GATA1* mutation in DS samples

DS neonates are at an increased risk of transient leukemia (TL) [49] and as many as 10% of them are affected by transient myeloid disorder [50]. Somatic mutations of *GATA1* exon 2 including insertions, deletions, and point mutations are found in almost all cases of DS–associated TL [51], [52]. *GATA1* encodes hematopoietic transcription factor [52], [53] and its mutation in DS samples could affect transcriptomes and lead to an unfair comparison between DS and controls. Therefore, we checked for the presence of *GATA1* mutation in DS neonates and children. Genomic DNAs from the DS samples were isolated with QIAamp Blood DNA Mini kits (Qiagen) according to the manufacturer's protocol. Amplicons including the whole *GATA1* exon 2 were obtained by PCR using forward (5′-TGTCTGAGGACCCCTTCTGT-3′) and reverse (5′-AAGCTTCCAGCCATTTCTGA-3′) primer and then sequenced. Sequence comparisons were performed by use of programs available from NCBI. However, no mutation of *GATA1* exon 2 was found in the DS samples used in this study (Files S 1).

### Array hybridization

For each sample, ribosomal RNA was removed from 1 µg of total RNA with the RiboMinus Human/Mouse Transcriptome Isolation kit (Invitrogen) [54], [55]. A similar approach for the depletion of abundant rRNA molecules was used in RNA-seq analysis [11], [56], which could investigate a plethora of non-polyadenilated mRNA. cDNA was synthesized using the GeneChip WT (Whole Transcripts) cDNA Synthesis and Amplification Kit (Affymetrix) according to manufacturer's instructions. The sense cDNA was fragmented and end labeled by use of the GeneChip WT Terminal Labeling Kit (Affymetrix). Approximately 5.5 mg of biotin-labeled DNA target was hybridized to the Affymetrix GeneChip Human Exon 1.0 ST Array at 45°C for 16 hr following manufacturer's protocol (Affymetrix). Arrays were washed after hybridization, stained on a GeneChip Fluidics Station 450 and scanned on a GCS3000 Scanner (Affymetrix). Raw data were extracted from the scanned images and the Expression Console software (Affymetrix) was used for data analysis.

### Normalization and summarization of array hybridization data

Expression Console software (Affymetrix) was used to quantile-normalize the probe fluorescence intensities over all 37 samples with PM-GCBG background correction. An iterative probe logarithmic intensity error (IterPlier) model (http://www.affymetrix.com/support/technical/technotes/plier_technote.pdf) was used to summarize the meta-probe set (representing gene expression) intensities. To stabilize variance, a constant of 16 was added to all probe set intensities, and then the signal values were log_2_ transformed. Removing the transcript clusters without any RefSeq-supported annotation, we generated the expression signals of 17,626 transcript clusters with core sets. A transcript cluster was considered to be reliably expressed when the log_2_-transformed expression signal was larger than 6.0 in at least 90% of the all samples in each group [54]. The remaining transcript clusters in each group, which met this criterion as determined by using R software (http://www.r-project.org/), were utilized for further analysis.

Raw microarray data have been deposited with GEO (Accession No. GSE35665).

### Identifying differentially expressed genes in each age group

In this study, gender was less well matched between DS and controls in each group. Gender and interaction of DS and gender effects could have an impact on gene expression variation. To identify differentially expressed genes between DS and control individuals in each group which are independent of the two effects, we used analysis of variance (ANOVA) at an individual gene level.

For each gene, the following linear model was used:
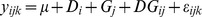
Where *y_ijk_* is the normalized expression of the gene in log2 for *i*th disease type (DS or control), *j*th gender (male or female), and *k*th sample in each group; the symbols *D*, *G* and *DG* represent the fixed effects due to the disease, gender as well as interaction of disease×gender, respectively; the error for each gene for sample *ijk* is designated as *εijk*. ANOVA was performed using R software. Multiple testing (BH correction) was performed on P values after ANOVA, no gene in DS neonates and only eight genes in DS children were significantly dysregulated (q<0.05). Thus, we referred to the method of Conti et al. [15] and applied P value after ANOVA and fold change (DS/control) for differentially expressed genes between DS and controls in each group. Specifically, genes with significantly different expression between DS and control individuals met following criteria: P value<0.01 and fold change (DS/control)>1.30 (up-regulated) or <0.77 (down-regulated). Here, fold change is equal to 2 ∧ delta mean value, which is the error of the mean of log_2_ (DS) and mean of log_2_ (control).

Due to broad age range (from 1 year to 13 years) in C group, we determined correlation of differentially expressed genes with age and assessed whether the variable had effects on gene expression. Pearson's correlation test was performed for age, as described by Lockstone et al [13] and P values were adjusted by use of BH correction.

### Quantitative real-time PCR

For validation of expression ratios between DS and control samples obtained from the array, total RNA was reverse transcribed into cDNA using PrimeScript™ RT reagent Kit (TaKaRa). Quantitative real-time PCR (QPCR) was performed on 11 genes from N group and 25 genes from C group using the LightCycler 480 (Roche Diagnostics) or the StepOne Plus (Applied Biosystems) systems with SYBR® Premix Ex Taq™ II (Perfect Real Time) (TaKaRa). Each gene was amplified in replicates of three. The genes and their primer sequences, designed by Primer 3 software [57], were described in Table S5. *UBA7* (NCBI Gene ID 7318) and *RNF4* (NCBI Gene ID 6047) were used as endogenous control genes [14]. For each sample, we normalized the mean cycle threshold value (C_t_) by subtracting the mean of the C_t_ generated from the two reference genes. The same ANOVA as the array analysis above was used to test statistical significance of difference in gene expression between DS and controls.

### Chromosomal distribution of significantly dysregulated genes in DS

Distribution of significantly dysregulated genes in DS individuals was tested against the null chromosomal distribution of the expressed transcript clusters in N and C group, respectively. Significant chromosomes were identified with binomial test (Pc<0.05 after BH correction), as described by Zhang et al [54]. STRIPE software was used to plot the chromosomal distribution of the dysregulated transcript clusters in each group [58].

### Cluster analysis

For the transcript clusters in each group that were differentially expressed between DS and control samples, Euclidean distance of expression levels was computed and hierarchical clustering was performed using complete-linkage method (the hclust function in the stats package) in R software. Heatmaps were plotted using the heatmap.2 function in the gplots package with the “scale = ‘row’” option set to “z-score normalize the rows”.

### GO and KEGG pathway analysis

Onto-Express and Pathway-Express in Onto-tools [59] were used to identify enriched Gene Ontology (GO) [60] terms and Kyoto Encyclopedia of Genes and Genomes (KEGG) [61] pathways, respectively. Among the differentially expressed genes in each group, GO terms or KEGG pathways that were over-represented relative to the gene set on the Affymetrix Human Exon 1.0 ST Array were selected (four or more hits [54], hypergeomatric test Pc<0.05 after BH correction ).

## Supporting Information

Figure S1 **Box plots of normalized expression levels of 36 genes from QPCR.** The Y-axis is normalized expression values and the x-axis is sample (D: DS versus C: control). Each panel represents a gene. DS and control in each panel is represented by red and green box in N group and by red and blue box in C group, respectively.
(TIF)

Figure S2 **Hierarchical clustering of the differentially expressed non-Hsa21 genes.** (A) hierarchical clustering of N group (DS: D11–D15 versus control: C16–C22) based on the non-Hsa21 dysregulated transcript clusters (rows). (B) hierarchical clustering of C group (DS: D1–D10 versus control: C1–C15) based on the non-Hsa21 differentially expressed transcript clusters.
(TIF)

Table S1 **List of the dysregulated genes in neonate group.**
(XLS)

Table S2 **List of the dysregulated genes in child group.**
(XLS)

Table S3 **Comparison between microarray data and QPCR.**
(DOC)

Table S4 **Characteristics of samples.**
(DOC)

Table S5 **List of oligonucleotide primers used in the QPCR.**
(DOC)

Table S6 **Expression pattern of known targets of the transcription factors.**
(DOC)

File S1 **Sequences of** ***GATA1*** **exon 2 in DS samples.**
(FASTA)
