# Supplementary figures and images for: Genome-Wide Expression Analysis in Down Syndrome: Insight into Immunodeficiency
Source: PLoS One. 2012 Nov 14;7(11):e49130. doi: 10.1371/journal.pone.0049130 (PMC3498323; doi:10.1371/journal.pone.0049130)

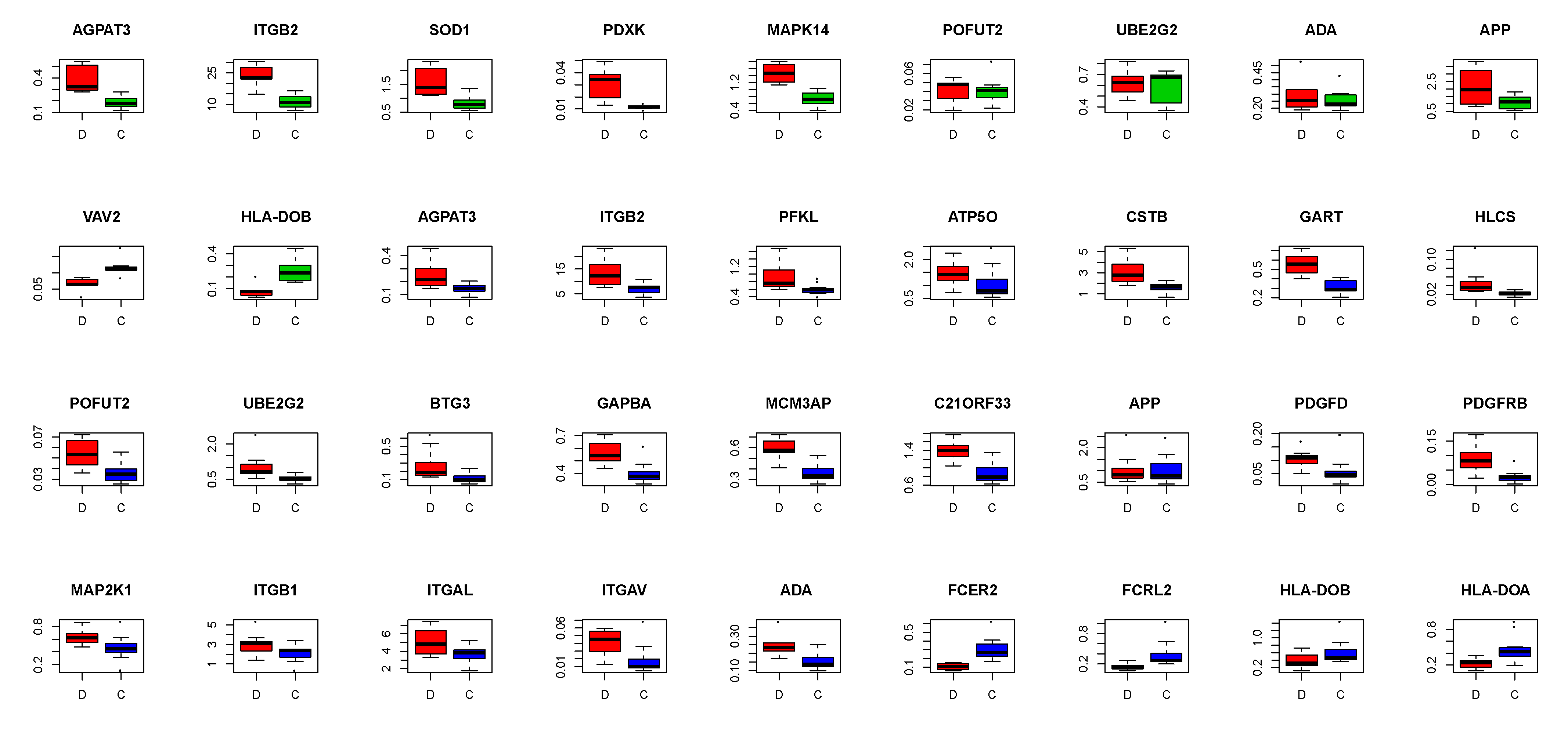

Supplement: Figure S1 — Box plots of normalized expression levels of 36 genes from QPCR. The Y-axis is normalized expression values and the x-axis is sample (D: DS versus C: control). Each panel represents a gene. DS and control in each panel is represented by red and green box in N group and by red and blue box in C group, respectively. (TIF) [file pone.0049130.s001.tif]

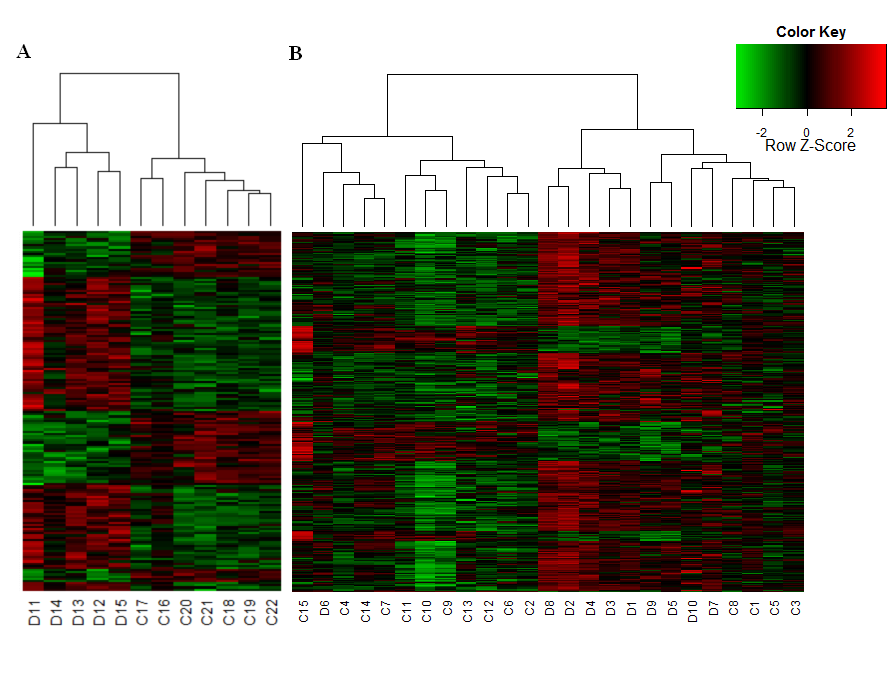

Supplement: Figure S2 — Hierarchical clustering of the differentially expressed non-Hsa21 genes. (A) hierarchical clustering of N group (DS: D11–D15 versus control: C16–C22) based on the non-Hsa21 dysregulated transcript clusters (rows). (B) hierarchical clustering of C group (DS: D1–D10 versus control: C1–C15) based on the non-Hsa21 differentially expressed transcript clusters. (TIF) [file pone.0049130.s002.tif]
