# Supplementary material for: Genome-Wide Expression Analysis in Down Syndrome: Insight into Immunodeficiency
Source: PLoS One. 2012 Nov 14;7(11):e49130. doi: 10.1371/journal.pone.0049130 (PMC3498323; doi:10.1371/journal.pone.0049130)
Supplement: Table S3 — Comparison between microarray data and QPCR. (DOC) [file pone.0049130.s005.doc]

**Table S3.** Comparison between microarray data and QPCR

| Age Group | Gene Symbol | Genebank Accession Number | DS/control Ratio by Array | Array Pvalue | DS/control  Ratio by QPCR | QPCR  Pvalue | Chromosome |
| --- | --- | --- | --- | --- | --- | --- | --- |
| N | *AGPAT3* | NM_020132 | 1.73 | 5.7E-03 | 2.08 | 5.9E-03 | Chr21 |
|  | *ITGB2* | NM_000211 | 2.03 | 1.9E-03 | 2.09 | 3.5E-03 | Chr21 |
|  | *PDXK* | NM_003681 | 2.51 | 5.2E-04 | 2.68 | 2.8E-03 | Chr21 |
|  | *SOD1* | NM_000454 | 2.40 | 8.3E-03 | 1.92 | 1.5E-02 | Chr21 |
|  | *MAPK14* | NM_001315 | 1.74 | 9.4E-04 | 2.01 | 1.4E-03 | Chr6 |
|  | *POFUT2* | NM_015227 | 0.92 | 0.62 | 0.98 | 0.92 | Chr21 |
|  | *APP* | NM_000484 | 1.85 | 0.17 | 1.98 | 7.3E-02 | Chr21 |
|  | *UBE2G2* | NM_182688 | 0.95 | 0.78 | 1.09 | 0.61 | Chr21 |
|  | *ADA* | NM_000022 | 1.16 | 0.59 | 1.13 | 0.59 | Chr20 |
|  | *HLA-DOB* | NM_002120 | 0.43 | 4.7E-03 | 0.33 | 3.1E-03 | Chr6 |
|  | *VAV2* | NM_001134398 | 0.51 | 3.9E-03 | 0.53 | 8.1E-03 | Chr9 |
| C | *AGPAT3* | NM_020132 | 1.79 | 1.0E-05 | 1.73 | 2.7E-03 | Chr21 |
|  | *ATP5O* | NM_001697 | 1.43 | 6.0E-03 | 1.42 | 4.8E-02 | Chr21 |
|  | *BTG3* | NM_001130914 | 1.99 | 8.5E-03 | 2.38 | 1.1E-03 | Chr21 |
|  | *C21orf33* | NM_004649 | 2.01 | 7.4E-04 | 1.61 | 2.5E-05 | Chr21 |
|  | *CSTB* | NM_000100 | 1.67 | 1.1E-03 | 1.96 | 4.5E-05 | Chr21 |
|  | *GABPA* | NM_002040 | 1.55 | 9.8E-03 | 1.42 | 3.6E-05 | Chr21 |
|  | *GART* | NM_000819 | 1.90 | 1.1E-03 | 1.80 | 9.2E-07 | Chr21 |
|  | *HLCS* | NM_000411 | 1.60 | 4.5E-03 | 2.14 | 4.9E-03 | Chr21 |
|  | *ITGB2* | NM_000211 | 1.73 | 3.5E-03 | 1.90 | 1.8E-04 | Chr21 |
|  | *MCM3AP* | NM_003906 | 1.63 | 2.6E-03 | 1.63 | 1.4E-06 | Chr21 |
|  | *PFKL* | NR_024108 | 1.74 | 3.1E-04 | 1.62 | 1.1E-03 | Chr21 |
|  | *POFUT2* | NM_015227 | 1.60 | 3.0E-04 | 1.53 | 1.3E-04 | Chr21 |
|  | *UBE2G2* | NM_182688 | 1.70 | 1.5E-03 | 1.93 | 2.5E-03 | Chr21 |
|  | *ITGB1* | NM_002211 | 1.57 | 7.8E-03 | 1.48 | 1.2E-02 | Chr10 |
|  | *PDGFD* | NM_025208 | 2.12 | 2.9E-03 | 1.86 | 3.9E-03 | Chr11 |
|  | *MAP2K1* | NM_002755 | 1.39 | 1.9E-04 | 1.40 | 1.7E-02 | Chr15 |
|  | *ITGAL* | NM_002209 | 1.65 | 1.7E-03 | 1.38 | 8.9E-03 | Chr16 |
|  | *ITGAV* | NM_002210 | 1.58 | 1.2E-04 | 2.42 | 2.4E-03 | Chr2 |
|  | *PDGFRB* | NM_002609 | 2.53 | 1.9E-04 | 3.43 | 1.4E-04 | Chr5 |
|  | *ADA* | NM_000022 | 1.72 | 1.3E-03 | 1.64 | 3.7E-04 | Chr20 |
|  | *APP* | NM_000484 | 1.27 | 0.14 | 1.01 | 0.95 | Chr21 |
|  | *FCRL2* | NM_030764 | 0.49 | 2.2E-04 | 0.39 | 7.3E-03 | Chr1 |
|  | *FCER2* | NM_002002 | 0.48 | 1.1E-04 | 0.36 | 4.2E-05 | Chr19 |
|  | *HLA-DOA* | NM_002119 | 0.61 | 7.5E-04 | 0.50 | 1.3E-04 | Chr6 |
|  | *HLA-DOB* | NM_002120 | 0.49 | 7.0E-05 | 0.63 | 2.5E-03 | Chr6 |
