# Supplementary material for: Genome-Wide Expression Analysis in Down Syndrome: Insight into Immunodeficiency
Source: PLoS One. 2012 Nov 14;7(11):e49130. doi: 10.1371/journal.pone.0049130 (PMC3498323; doi:10.1371/journal.pone.0049130)
Supplement: Table S4 — Characteristics of samples. (DOC) [file pone.0049130.s006.doc]

**Table S4. Characteristics of samples**

| Age group | Sample ID | Sex | Age |
| --- | --- | --- | --- |
| Child group ^a^ | C1 | Female | 11 years |
|  | C2 | Male | 13 years |
|  | C3 | Male | 12 years |
|  | C4 | Male | 12 years |
|  | C5 | Male | 12 years |
|  | C6 | Male | 9 years |
|  | C7 | Female | 9 years |
|  | C8 | Male | 8 years |
|  | C9 | Female | 4 years |
|  | C10 | Male | 5 years |
|  | C11 | Male | 5 years |
|  | C12 | Female | 3 years |
|  | C13 | Female | 3 years |
|  | C14 | Male | 2.5 years |
|  | C15 | Male | 2 years |
|  | D1 | Female | 11 years |
|  | D2 | Male | 11 years |
|  | D3 | Male | 11 years |
|  | D4 | Male | 6 years |
|  | D5 | Male | 5 years |
|  | D6 | Female | 4 years |
|  | D7 | Female | 2 years |
|  | D8 | Female | 2 years |
|  | D9 | Male | 2 years |
|  | D10 | Male | 1 years |
| Neonate group ^b^ | C16 | Male | 3 days |
|  | C17 | Male | 7 days |
|  | C18 | Female | 30 days |
|  | C19 | Male | 23 days |
|  | C20 | Male | 28 days |
|  | C21 | Female | 38 days |
|  | C22 | Female | 23 days |
|  | D11 | Male | 7 days |
|  | D12 | Female | 10 days |
|  | D13 | Female | 7 days |
|  | D14 | Female | 11 days |
|  | D15 | Female | 30 days |

^a^ peripheral blood mononuclear cells (PBMCs)

^b^ peripheral blood cells (PBCs)
