# Supplementary material for: Genome-Wide Expression Analysis in Down Syndrome: Insight into Immunodeficiency
Source: PLoS One. 2012 Nov 14;7(11):e49130. doi: 10.1371/journal.pone.0049130 (PMC3498323; doi:10.1371/journal.pone.0049130)
Supplement: Table S5 — List of oligonucleotide primers used in the QPCR. (DOC) [file pone.0049130.s007.doc]

**Table S5. List of oligonucleotide primers used in the QPCR**

| Primer | Sequence (5’-3’) | Age Group |
| --- | --- | --- |
| *PDXK_UP* | TGTCCCCTTCCATCTCTGAC | N |
| *PDXK_LOW* | GAAGGCATATCCCACCAGAA | N |
| *SOD1_UP* | AGGGCATCATCAATTTCGAGC | N |
| *SOD1_LOW* | GCCCACCGTGTTTTCTGGA | N |
| *MAPK14_UP* | GGGGCAGATCTGAACAACAT | N |
| *MAPK14_LOW* | CCACGTAGCCTGTCATTTCA | N |
| *VAV2_UP* | TCAAGGTGCATCACAGCTTC | N |
| *VAV2_LOW* | TTCAGTGTGCACTCCTCGAC | N |
| *ATP5O_UP* | CAGGTGCGATGCTTCAGTACC | C |
| *ATP5O_LOW* | CCACTTTGGGTTCCTTCAGG | C |
| *BTG3_UP* | TCCTCCTCCTGTTCCATTTG | C |
| *BTG3_LOW* | AATCCAGTGATTCCGGTCAC | C |
| *C21orf33_UP* | CGCAGAGAATAAACGCCTTC | C |
| *C21orf33_LOW* | ACCAACGCTACCATCACTCC | C |
| *CSTB_UP* | TGTCATTCAAGAGCCAGGTG | C |
| *CSTB_LOW* | AGCTCATCATGCTTGGCTTT | C |
| *GABPA_UP* | GCATGTGTGCCTGTTTCATC | C |
| *GABPA_LOW* | CACTGCTCTTTTGCCAGTCA | C |
| *GART_UP* | CCCTCACCTGGAAAGAATCA | C |
| *GART_LOW* | CTGGTCACCACAACCATCAG | C |
| *HLCS_UP* | GTCGTGACTGTGCTGGAGAA | C |
| *HLCS_LOW* | AACCTGGAGGAAGCCAGAAT | C |
| *MCM3AP_UP* | GAGAGGGACGTTTGGCAATA | C |
| *MCM3AP_LOW* | TCCCCTCTTGAGCACACTCT | C |
| *PFKL_UP* | GGAGCTTCGAGAACAACTGG | C |
| *PFKL_LOW* | CTGTGTGTCCATGGGAGATG | C |
| *ITGB1_UP* | ATCTGCGAGTGTGGTGTCTG | C |
| *ITGB1_LOW* | GGGGTAATTTGTCCCGACTT | C |
| *PDGFD_UP* | GTGGAGGAAATTGTGGCTGT | C |
| *PDGFD_LOW* | CGTTCATGGTGATCCAACTG | C |
| *MAP2K1_UP* | ATGTCAAGCCCTCCAACATC | C |
| *MAP2K1_LOW* | GGCGACATGTAGGACCTTGT | C |
| *ITGAL_UP* | AGAGTCCAGGCTTCTGTCCA | C |
| *ITGAL_LOW* | GGATGGGGATGATGGTAGTG | C |
| *ITGAV_UP* | CTGGCTTAAGAGGGCTGTGA | C |
| *ITGAV_LOW* | TGGGTAGTGGCTGCACATAG | C |
| *PDGFRB_UP* | CCTGCTATGAGGCTTTGGAG | C |
| *PDGFRB_LOW* | GACAAATGTGCAACCACCTG | C |
| *FCRL2_UP* | GCTCAGCAAACATCAGGACA | C |
| *FCRL2_LOW* | GCAGGTGATAAGCCTCAAGC | C |
|  |  | (continued) |

**Table S5.** (continued)

| Primer | Sequence (5’-3’) | Age Group |
| --- | --- | --- |
| *HLA-DOA_UP* | AACCCTCACAACCCACAGAG | C |
| *HLA-DOA_LOW* | AACTACACAGGGCTGGGATG | C |
| *AGPAT3_UP* | TCAACCACAACTTCGAGATCG | N & C |
| *AGPAT3_LOW* | TACTCGGGGTAGTCCGACAG | N & C |
| *ITGB2_UP* | CAACGTATGCGAGTGCCATTC | N & C |
| *ITGB2_LOW* | TTCACGGGGTTGTTCGACAG | N & C |
| *POFUT2_UP* | CAAAGAGGGCAACGCTAGAC | N & C |
| *POFUT2_LOW* | AGATGTGGAAAGTGGCTGCT | N & C |
| *APP_UP* | TGGCCCTGGAGAACTACATC | N & C |
| *APP_LOW* | AATCACACGGAGGTGTGTCA | N & C |
| *UBE2G2_UP* | GATGACCGGGAGCAGTTCTA | N & C |
| *UBE2G2_LOW* | TTGCAAGTCTGCCTTGTTTG | N & C |
| *ADA_UP* | GGGACATGGGCTTTACTGAA | N & C |
| *ADA_LOW* | GAGGAGTGGCGTCTTCAGAG | N & C |
| *HLA-DOB_UP* | TGTCTACACCTGCCTTGTCG | N & C |
| *HLA-DOB_LOW* | ATGACGATTCCCACCAGAAG | N & C |
| *UBA7F ^a^_UP* | GAGATGTCAGAACTACGGGATT | N & C |
| *UBA7R ^a^_LOW* | GGCGAAAGGCACTACGAG | N & C |
| *RNF4F ^a^_UP* | GAAGGAGCAGGGACTCAGCA | N & C |
| *RNF4R ^a^_LOW* | ACTAACTGGAAGTGAGCCAAACA | N & C |

^a^ endogenous gene
