# Supplementary material for: Genome-Wide Expression Analysis in Down Syndrome: Insight into Immunodeficiency
Source: PLoS One. 2012 Nov 14;7(11):e49130. doi: 10.1371/journal.pone.0049130 (PMC3498323; doi:10.1371/journal.pone.0049130)
Supplement: Table S6 — Expression pattern of known targets of the transcription factors. (DOC) [file pone.0049130.s008.doc]

Table S6. Expression pattern of known targets of the transcription factors

| Group | Transcript factor | regulate | target | FC^b^ | P (ANOVA) | Reference |
| --- | --- | --- | --- | --- | --- | --- |
| C | *GFI1* | repress | *CDKN2B* | 1.07 | 0.45 | [5] |
|  |  | repress | *SPI1* | 0.96 | 0.88 | [6] |
|  |  | repress | *ID2* | 1.13 | 0.36 | [7] |
|  |  | repress | *CDKN1A* | 0.93 | 0.80 | [8] |
|  |  | repress | *BAX* | 1.01 | 0.91 | [9] |
|  | *HSF4* | repress | *HIF1A* | 0.85 | 0.51 | [10] |
|  | *SPIB* | activate | *GRAP2* | 1.10 | 0.52 | [11] |
|  |  | activate | *P2RY10* | 0.70 | 0.04 | [12] |
|  |  | activate | *BTK* | 0.89 | 0.44 | [13] |
|  | *RUNX1* | repress | *SERPINB13* | 1.08 | 0.32 | [14] |
|  |  | repress | *LAT2* | 0.87 | 0.42 | [15] |
|  |  | activate | *PRKCQ* | 1.56 | 0.02 | [16] |
|  |  | activate | *MYL9* | 1.50 | 0.40 | [17] |
|  |  | activate | *ALOX12* | 1.08 | 0.42 | [18] |
|  |  | activate | *PRKACB* | 0.95 | 0.80 | [19] |
|  | *GABPA* | activate | *MTERF* | 1.05 | 0.75 | [20] |
|  |  | activate | *POLRMT* | 1.16 | 0.03 | [20] |
|  |  | activate | *SSBP1* | 0.90 | 0.71 | [20] |
|  |  | activate | *MTIF2* | 1.11 | 0.58 | [21] |
|  | *PAX5* | activate | *CD19* | 0.63 | 0.01 | [2,3,22,23] |
|  |  | activate | *CD79A* | 0.45 | 5.1E-05 | [2,3,23,24] |
|  |  | activate | *BLNK* | 0.55 | 4.2E-03 | [2,3,23] |
|  |  | activate | *EBF1* | 0.54 | 0.01 | [2,3,23] |
|  |  | activate | *SPIB* | 0.49 | 9.0E-04 | [2,3] |
|  |  | activate | *FCER2* | 0.48 | 1.1E-04 | [4] |
|  | *IKZF4* | activate | *DLG4* | 0.90 | 0.51 | [25] |
|  | *KLF13* | activate | *CCL5* | 1.48 | 0.02 | [26,27] |
|  | *MAF* | repress | *ANPEP* | 0.94 | 0.83 | [28,29] |
|  |  | activate | *CCND2* | 1.22 | 0.18 | [30,31] |
|  |  | activate | *ITGB7* | 1.72 | 0.01 | [31] |
|  |  | repress | *BCL2* | 1.35 | 0.24 | [32,33] |
|  |  | activate | *CASP9* | 1.00 | 0.96 | [34] |
|  |  | activate | *CTGF* | 1.08 | 0.29 | [35] |
|  |  | activate | *CCR1* | 1.03 | 0.92 | [31] |
|  |  | activate | *IL4* | - ^a^ | - | [36,37] |
|  |  | activate | *IL10* | - ^a^ | - | [38] |
| N | *GRHL1* | repress | *CYP11A1* | - ^a^ | - | [39] |
|  | *PAX5* | activate | *CD19* | 0.59 | 0.02 | [2,3,22,23] |
|  |  | activate | *CD79A* | 0.41 | 0.06 | [2,3,23,24] |
|  |  | activate | *BLNK* | 0.47 | 0.04 | [2,3,23] |
|  |  | activate | *EBF1* | - ^a^ | - | [2,3,23] |
|  |  | activate | *SPIB* | - ^a^ | - | [2,3] |
|  |  | activate | *FCER2* | - ^a^ | - | [4] |

^a^ data not detected in this study

^b^ fold change (DS/control)

1. FitzPatrick DR (2005) Transcriptional consequences of autosomal trisomy: primary gene dosage with complex downstream effects. Trends Genet 21: 249-253.
2. Schebesta A, McManus S, Salvagiotto G, Delogu A, Busslinger GA, et al. (2007) Transcription factor Pax5 activates the chromatin of key genes involved in B cell signaling, adhesion, migration, and immune function. Immunity 27: 49-63.
3. Pridans C, Holmes ML, Polli M, Wettenhall JM, Dakic A, et al. (2008) Identification of Pax5 target genes in early B cell differentiation. J Immunol 180: 1719-1728.
4. Visan I, Goller M, Berberich I, Kneitz C, Tony HP (2003) Pax-5 is a key regulator of the B cell-restricted expression of the CD23a isoform. Eur J Immunol 33: 1163-1173.
5. Basu S, Liu Q, Qiu Y, Dong F (2009) Gfi-1 represses CDKN2B encoding p15INK4B through interaction with Miz-1. Proc Natl Acad Sci U S A 106: 1433-1438.
6. Khanna-Gupta A, Sun H, Zibello T, Lee HM, Dahl R, et al. (2007) Growth factor independence-1 (Gfi-1) plays a role in mediating specific granule deficiency (SGD) in a patient lacking a gene-inactivating mutation in the C/EBPepsilon gene. Blood 109: 4181-4190.
7. Li H, Ji M, Klarmann KD, Keller JR (2010) Repression of Id2 expression by Gfi-1 is required for B-cell and myeloid development. Blood 116: 1060-1069.
8. Liu Q, Basu S, Qiu Y, Tang F, Dong F (2010) A role of Miz-1 in Gfi-1-mediated transcriptional repression of CDKN1A. Oncogene 29: 2843-2852.
9. Grimes HL, Gilks CB, Chan TO, Porter S, Tsichlis PN (1996) The Gfi-1 protooncoprotein represses Bax expression and inhibits T-cell death. Proc Natl Acad Sci U S A 93: 14569-14573.
10. Chen R, Liliental JE, Kowalski PE, Lu Q, Cohen SN (2011) Regulation of transcription of hypoxia-inducible factor-1alpha (HIF-1alpha) by heat shock factors HSF2 and HSF4. Oncogene 30: 2570-2580.
11. Garrett-Sinha LA, Hou P, Wang D, Grabiner B, Araujo E, et al. (2005) Spi-1 and Spi-B control the expression of the Grap2 gene in B cells. Gene 353: 134-146.
12. Rao S, Garrett-Sinha LA, Yoon J, Simon MC (1999) The Ets factors PU.1 and Spi-B regulate the transcription in vivo of P2Y10, a lymphoid restricted heptahelical receptor. J Biol Chem 274: 34245-34252.
13. Muller S, Sideras P, Smith CI, Xanthopoulos KG (1996) Cell specific expression of human Bruton's agammaglobulinemia tyrosine kinase gene (Btk) is regulated by Sp1- and Spi-1/PU.1-family members. Oncogene 13: 1955-1964.
14. Boyapati A, Ren B, Zhang DE (2011) SERPINB13 is a novel RUNX1 target gene. Biochem Biophys Res Commun 411: 115-120.
15. Duque-Afonso J, Solari L, Essig A, Berg T, Pahl HL, et al. (2011) Regulation of the adaptor molecule LAT2, an in vivo target gene of AML1/ETO (RUNX1/RUNX1T1), during myeloid differentiation. Br J Haematol 153: 612-622.
16. Jalagadugula G, Mao G, Kaur G, Dhanasekaran DN, Rao AK (2011) Platelet protein kinase C-theta deficiency with human RUNX1 mutation: PRKCQ is a transcriptional target of RUNX1. Arterioscler Thromb Vasc Biol 31: 921-927.
17. Jalagadugula G, Mao G, Kaur G, Goldfinger LE, Dhanasekaran DN, et al. (2010) Regulation of platelet myosin light chain (MYL9) by RUNX1: implications for thrombocytopenia and platelet dysfunction in RUNX1 haplodeficiency. Blood 116: 6037-6045.
18. Kaur G, Jalagadugula G, Mao G, Rao AK (2010) RUNX1/core binding factor A2 regulates platelet 12-lipoxygenase gene (ALOX12): studies in human RUNX1 haplodeficiency. Blood 115: 3128-3135.
19. Hug BA, Ahmed N, Robbins JA, Lazar MA (2004) A chromatin immunoprecipitation screen reveals protein kinase Cbeta as a direct RUNX1 target gene. J Biol Chem 279: 825-830.
20. Bruni F, Polosa PL, Gadaleta MN, Cantatore P, Roberti M (2010) Nuclear respiratory factor 2 induces the expression of many but not all human proteins acting in mitochondrial DNA transcription and replication. J Biol Chem 285: 3939-3948.
21. Hayashi R, Ueda T, Farwell MA, Takeuchi N (2007) Nuclear respiratory factor 2 activates transcription of human mitochondrial translation initiation factor 2 gene. Mitochondrion 7: 195-203.
22. Kozmik Z, Wang S, Dorfler P, Adams B, Busslinger M (1992) The promoter of the CD19 gene is a target for the B-cell-specific transcription factor BSAP. Mol Cell Biol 12: 2662-2672.
23. McManus S, Ebert A, Salvagiotto G, Medvedovic J, Sun Q, et al. (2011) The transcription factor Pax5 regulates its target genes by recruiting chromatin-modifying proteins in committed B cells. EMBO J 30: 2388-2404.
24. Fitzsimmons D, Hodsdon W, Wheat W, Maira SM, Wasylyk B, et al. (1996) Pax-5 (BSAP) recruits Ets proto-oncogene family proteins to form functional ternary complexes on a B-cell-specific promoter. Genes Dev 10: 2198-2211.
25. Bao J, Lin H, Ouyang Y, Lei D, Osman A, et al. (2004) Activity-dependent transcription regulation of PSD-95 by neuregulin-1 and Eos. Nat Neurosci 7: 1250-1258.
26. Huang B, Ahn YT, McPherson L, Clayberger C, Krensky AM (2007) Interaction of PRP4 with Kruppel-like factor 13 regulates CCL5 transcription. J Immunol 178: 7081-7087.
27. Song A, Patel A, Thamatrakoln K, Liu C, Feng D, et al. (2002) Functional domains and DNA-binding sequences of RFLAT-1/KLF13, a Kruppel-like transcription factor of activated T lymphocytes. J Biol Chem 277: 30055-30065.
28. Hedge SP, Kumar A, Kurschner C, Shapiro LH (1998) c-Maf interacts with c-Myb to regulate transcription of an early myeloid gene during differentiation. Mol Cell Biol 18: 2729-2737.
29. Mahoney KM, Petrovic N, Schacke W, Shapiro LH (2007) CD13/APN transcription is regulated by the proto-oncogene c-Maf via an atypical response element. Gene 403: 178-187.
30. Morito N, Yoh K, Fujioka Y, Nakano T, Shimohata H, et al. (2006) Overexpression of c-Maf contributes to T-cell lymphoma in both mice and human. Cancer Res 66: 812-819.
31. Hurt EM, Wiestner A, Rosenwald A, Shaffer AL, Campo E, et al. (2004) Overexpression of c-maf is a frequent oncogenic event in multiple myeloma that promotes proliferation and pathological interactions with bone marrow stroma. Cancer Cell 5: 191-199.
32. Hegde SP, Zhao J, Ashmun RA, Shapiro LH (1999) c-Maf induces monocytic differentiation and apoptosis in bipotent myeloid progenitors. Blood 94: 1578-1589.
33. Peng S, Lalani S, Leavenworth JW, Ho IC, Pauza ME (2007) c-Maf interacts with c-Myb to down-regulate Bcl-2 expression and increase apoptosis in peripheral CD4 cells. Eur J Immunol 37: 2868-2880.
34. Peng S, Wu H, Mo YY, Watabe K, Pauza ME (2009) c-Maf increases apoptosis in peripheral CD8 cells by transactivating Caspase 6. Immunology 127: 267-278.
35. Omoteyama K, Ikeda H, Imaki J, Sakai M (2006) Activation of connective tissue growth factor gene by the c-Maf and Lc-Maf transcription factors. Biochem Biophys Res Commun 339: 1089-1097.
36. Kim JI, Ho IC, Grusby MJ, Glimcher LH (1999) The transcription factor c-Maf controls the production of interleukin-4 but not other Th2 cytokines. Immunity 10: 745-751.
37. Chen XP, Falkner DH, Morel PA (2005) Impaired IL-4 production by CD8+ T cells in NOD mice is related to a defect of c-Maf binding to the IL-4 promoter. Eur J Immunol 35: 1408-1417.
38. Xu J, Yang Y, Qiu G, Lal G, Wu Z, et al. (2009) c-Maf regulates IL-10 expression during Th17 polarization. J Immunol 182: 6226-6236.
39. Henderson YC, Frederick MJ, Jayakumar A, Choi Y, Wang MT, et al. (2007) Human LBP-32/MGR is a repressor of the P450scc in human choriocarcinoma cell line JEG-3. Placenta 28: 152-160.
40. Slonim DK, Koide K, Johnson KL, Tantravahi U, Cowan JM, et al. (2009) Functional genomic analysis of amniotic fluid cell-free mRNA suggests that oxidative stress is significant in Down syndrome fetuses. Proc Natl Acad Sci U S A 106: 9425-9429.
41. Bruce KD, Cagampang FR, Argenton M, Zhang J, Ethirajan PL, et al. (2009) Maternal high-fat feeding primes steatohepatitis in adult mice offspring, involving mitochondrial dysfunction and altered lipogenesis gene expression. Hepatology 50: 1796-1808.
